# Elemental Content of Calcium Oxalate Stones from a Canine Model of Urinary Stone Disease

**DOI:** 10.1371/journal.pone.0128374

**Published:** 2015-06-11

**Authors:** David W. Killilea, Jodi L. Westropp, Ryoji Shiraki, Matthew Mellema, Jennifer Larsen, Arnold J. Kahn, Pankaj Kapahi, Thomas Chi, Marshall L. Stoller

**Affiliations:** 1 Nutrition & Metabolism Center, Children’s Hospital Oakland Research Institute, Oakland, California, United States of America; 2 Medicine and Epidemiology, UC Davis School of Veterinary Medicine, Davis, California, United States of America; 3 Surgical & Radiological Sciences, UC Davis School of Veterinary Medicine, Davis, California, United States of America; 4 Molecular Biosciences, UC Davis School of Veterinary Medicine, Davis, California, United States of America; 5 Buck Institute for Research in Aging, Novato, California, United States of America; 6 Department of Urology, University of California San Francisco, San Francisco, California, United States of America; Emory University, UNITED STATES

## Abstract

One of the most common types of urinary stones formed in humans and some other mammals is composed of calcium oxalate in ordered hydrated crystals. Many studies have reported a range of metals other than calcium in human stones, but few have looked at stones from animal models such as the dog. Therefore, we determined the elemental profile of canine calcium oxalate urinary stones and compared it to reported values from human stones. The content of 19 elements spanning 7-orders of magnitude was quantified in calcium oxalate stones from 53 dogs. The elemental profile of the canine stones was highly overlapping with human stones, indicating similar inorganic composition. Correlation and cluster analysis was then performed on the elemental profile from canine stones to evaluate associations between the elements and test for potential subgrouping based on elemental content. No correlations were observed with the most abundant metal calcium. However, magnesium and sulfur content correlated with the mineral hydration form, while phosphorous and zinc content correlated with the neuter status of the dog. Inter-elemental correlation analysis indicated strong associations between barium, phosphorous, and zinc content. Additionally, cluster analysis revealed subgroups within the stones that were also based primarily on barium, phosphorous, and zinc. These data support the use of the dog as a model to study the effects of trace metal homeostasis in urinary stone disease.

## Introduction

Symptomatic urinary stone disease (urolithiasis) is a source of severe pain and infection with growing incidence and penetrance worldwide [1]. In the US alone, treatment for urinary stones accounts for more than $2 billion in annual medical expenditures, yet there has been little progress in the development of preventative strategies for stone management even after several decades of investigation. More insight into the pathophysiology of stone formation is needed to drive effective novel treatments for this disease [2]. One approach is to utilize animal models to study urolithiasis. The dog has generated research interest because it spontaneously forms urinary stones that physically and chemically resemble those found in humans [3]. Like the human disease, urolithiasis in dogs results in significant morbidity and veterinary costs, and knowledge of etiology is lacking. The most common urolith submitted to clinical laboratories in the past 15 years from dogs is composed predominantly of calcium oxalate (CaOx), as in humans. CaOx stones can occur in the upper or lower urinary tract in dogs, and treatment modalities used are similar to those used in humans, including laser lithotripsy and dietary modification [4–10]. In human calcium-based stones, several studies have reported the presence of proteins, organic acids, polysaccharides, and a variety of metals other than calcium, revealing a complex chemistry in the stones [11–16]. However, compositional work with similar type stones in dogs has not been as well developed.

In CaOx stones, the mineral type is classified based on hydration state, namely as a monohydrate (CaC_2_O_4_·H_2_O, whewellite, or COM) or a dihydrate (CaC_2_O_4_·2H_2_O, weddellite, or COD), as the major groups. Understanding the subtype of CaOx mineral is important because it affects clinical treatment. For example, stones composed primarily of COD are known to be more sensitive to extracorporeal shock wave lithotripsy (SWL) than other calcium mineral types, directing a more invasive but often more successful surgical approach [17–19]. Determining the composition of these CaOx subtypes requires specialized equipment and training, and has a high error rate. Furthermore, these conventional techniques cannot identify the other minor components within the stones that might have functional relevance. Having more robust compositional data on urolith composition may be useful to the clinician for therapeutic and preventative strategies. It is known that different calcium-based minerals have varying capacity to incorporate trace metals [11,13,16], so the elemental profile might also be useful for identifying the subtypes of CaOx stones. This study will measure the elemental profile in the common CaOx stone type in dogs, as an emerging animal model for studying urinary stone disease. The compositional metal profile will then be compared to the known metal content in human CaOx stones and to other stone characteristics in the dog.

## Methods

### Veterinary information

Canine CaOx urinary stones submitted the Gerald V. Ling Urinary Stone Analysis Laboratory at the UC Davis School of Veterinary Medicine (http://www.vetmed.ucdavis.edu/usal) between 2013–2014 were used for this study. The uroliths were chosen at random from a collection of stones stored at the laboratory. Dog and stone information was obtained from a relational database originating from questionnaires provided by the submitting veterinarians that included age, breed, sex, body weight, and current diet(s) of the dog. Institutional Animal Care and Use Committee approval was not needed for use of the urinary stones since the removal of the stones was determined to be medically necessary. Stone sample analysis is part of standard medical care, at which time they became the property of the laboratory and available for this study.

### Determination of stone chemistry

Mineral analysis of the canine stones was determined as previously described [3,20]. Calculi are examined for surface and cross sectional textures under a dissecting microscope. Mineral identify was determined using polarized light microscopy and infrared spectroscopy based on crystallographic features such as refractive index, birefringence, and crystal shape. For purposes of this study, CaOx stones included COM, COD, or a combination of both; minor levels of apatite (Ca_5_(PO_4_)_3_(F,Cl,OH)) or brushite (CaHPO_4_·2H_2_O) were accepted if 1% or less. The percentage of the detected mineral within each layer was estimated by calculating a mean value for the crystal counts obtained by microscopic examination of 5–10 microscopic slides.

### Determination of stone elemental content

The elemental content of the canine stone samples was determined by inductively-coupled plasma optical emission spectrometry (ICP-OES) at the CHORI Elemental Analysis Facility (http://www.chori.org/Services/Elemental_Analysis_Facility/elemental_analysis.html). For each stone, two separate fragments from a single stone sample were randomly selected for elemental analysis. The mean stone fragment size was 98 ± 31 mg. Selected stone samples were then digested in 0.5 ml 70% HNO_3_ and incubated overnight at 60°C with 150–200 rpm orbital shaking. The acid lysates were then diluted to 5% HNO_3_ with water, clarified by centrifugation, and introduced via a pneumatic concentric nebulizer using argon carrier gas into a Vista Pro ICP-OES (Varian, Inc). The ICP-OES was calibrated using National Institute of Standards and Technology-traceable elemental standards and reference material. The utilized method measured 34 elements with a minimum detection between 0.031–3.125 parts per million (S1 Table). Instrument precision for each element was typically between 5–10%. Cesium (50 ppm) was used for ionization suppression and yttrium (5 ppm) was used as an internal standard for all samples. All reagents and plasticware were certified or routinely tested for trace metal work. Elemental content data was summarized using native software (ICP Expert; Varian Inc) and normalized to the weight of the stone samples.

### Statistics

Graph and standard statistical testing was conducted using Prism 6c (GraphPad Software, Inc.) Outlier analysis was conducted using the GraphPad ROUT algorithm with Q = 0.1% [21]. Correlations between element values and stone characteristics were tested using t-tests or one-way, two-tailed ANOVA with Tukey’s post hoc test as appropriate; statistical significance was defined as *p*<0.05. Correlations between element values were tested using a simple linear regression algorithm, where goodness of fit was reported using coefficient of determination (r^2^) values. Cluster analysis of elemental content was conducted using Cluster 3.0 (Michael Eisen, Stanford University and Michiel de Hoon, University of Tokyo) and TreeView 1.1.6r4 (Alok Saldanha, Free Software Foundation, Inc.) [22].

## Results

**Fig 1:**
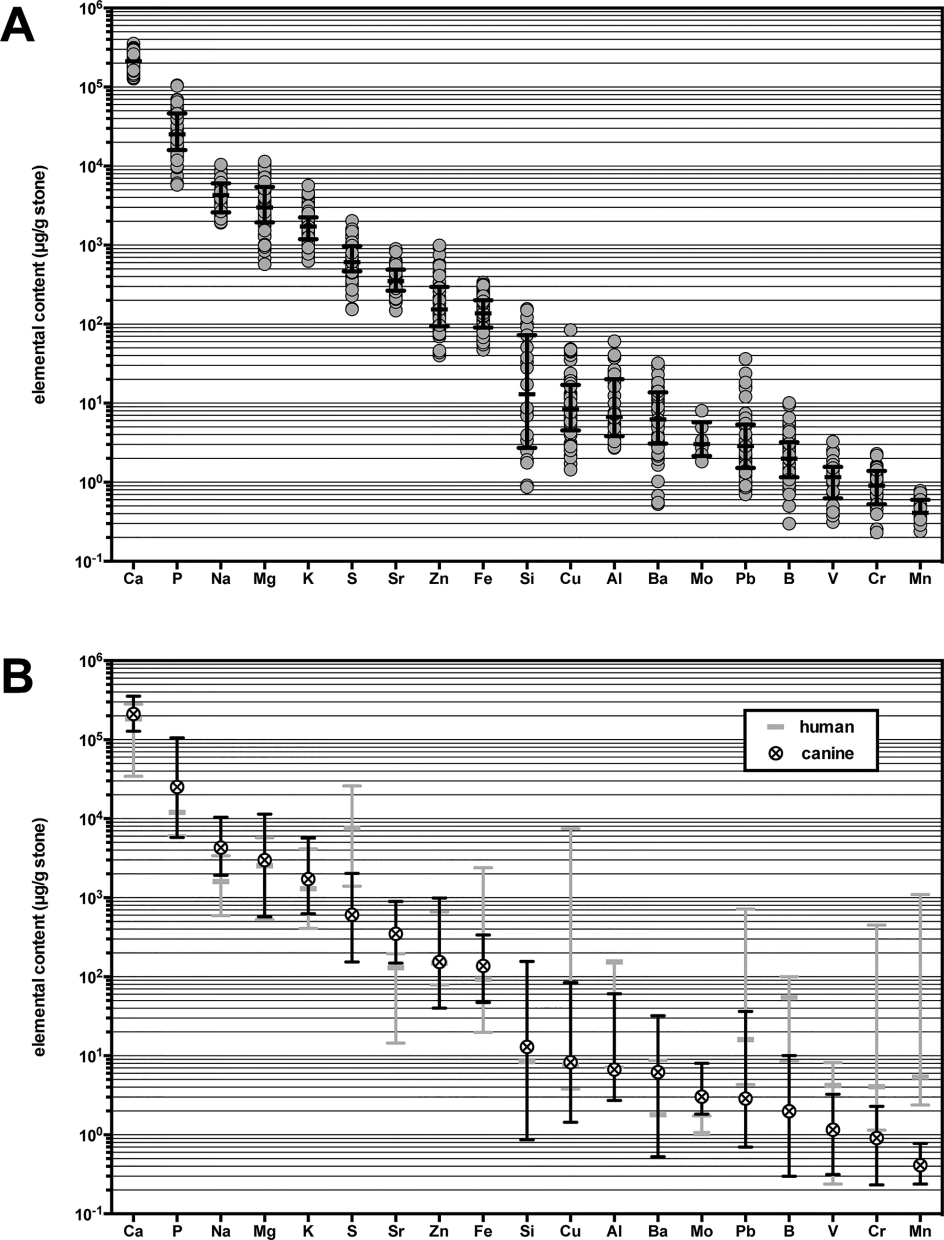
Rank order of weight-normalized elemental content in canine calcium oxalate-containing urinary stones. (A) The content of 19 elements (n = 6–53) quantified from verified CaOx stones types from canine patients is summarized and ordered based on abundance. Each data point represents the average value of two replicate stone or stone fragments from a single canine (gray circle). Superimposed on the data points is the group median ± the interquartile range (middle and error bars). Detectable elements are listed in rank order based on group median. (B) The median ± range of elemental content from canine CaOx stones (black symbol and error bars) is superimposed on the median ± range of elemental content reported from 4 studies [23–26] of human CaOx stones (gray middle and error bars) following the same rank order.

Fifty-three canine stone samples were randomly selected from a biobank of dog urinary stones identified as predominantly CaOx. Physical information for the dog and stone type was available for most of these dogs, showing a wide range of breeds and other characteristics (Table 1). Each canine stone sample was then analyzed for elemental content. Elemental values were included only if detectable in at least 10% of all stone samples, though most were detectable in >90% of stone samples. Nineteen elements were quantified, including aluminum (Al), barium (Ba), boron (B), calcium (Ca), chromium (Cr), copper (Cu), iron (Fe), lead (Pb), magnesium (Mg), manganese (Mn), molybdenum (Mo), phosphorous (P), potassium (K), silicon (Si), sodium (Na), strontium (Sr), sulfur (S), vanadium (V), and zinc (Zn). Five other elements including lithium (Li), rubidium (Rb), selenium (Se), tin (Sn), and titanium (Ti) were detectable in fewer than 10% of the stone samples, so were not included in this analysis. From this dataset, outlier analysis was applied which removed between 0–8 values from each elemental category. The result was a rank-ordered profile of weight-normalized element components spanning 7-orders of magnitude in concentration within the canine CaOx stones (Fig 1A and S2 Table). The range and variability of elemental content within the canine stones was then compared to the reported elemental content in human CaOx stone types (Fig 1B). Four studies have been published in the last 5 years that provide elemental values for predominantly CaOx stones from human patients with active urinary stone disease [23–26]. There was a high degree of overlap in the content of the constitutive and trace element groups between human and dog stones, indicating chemical similarity of inorganic component in these stones.

**Table 1:** Physiological characteristics of the dogs with urinary stones used in this study.

ID	BREED	AGE AT TIME OF STONE	SEX	STATUS	WEIGHT	STONE SURFACE (% COM/ % COD/ % APA)	STONE CORE (% COM/ % COD/ % APA)
1	Bichon Frise	8	female	spay	5.1	0% / 100% / 0%	100% / 0% / 0%
2	X-Pembroke Welsh Corgi	9	male	neutered	14.8	100% / 0% / 0%	100% / 0% / 0%
3	Yorkshire Terrier	10	male	neutered	3.6	100% / 0% / 0%	100% / 0% / 0%
4	X-Border Collie	12	female	intact	29.0	0% / 100% / 0%	20% / 80% / 0%
5	X-Chihuahua	11	male	neutered	6.2	10% / 90% / 0%	100% / 0% / 0%
6	Poodle, Miniature	11	male	intact	3.3	10% / 90% / 0%	100% / 0% / 0%
7	Shih Tzu	14	female	spay	NA	100% / 0% / 0%	100% / 0% / 0%
8	Golden Retriever	11	male	neutered	33.5	0% / 100% / 0%	99% / 1% / 0%
9	Poodle, Miniature	15	female	spay	8.0	0% / 100% / 0%	5% / 95% / 0%
10	Dachshund	12	female	spay	0.0	5% / 95% / 0%	100% / 0% / 0%
11	Shih Tzu	10	male	neutered	7.9	0% / 100% / 0%	100% / 0% / 0%
12	X-Pomeranian	11	male	neutered	4.1	0% / 100% / 0%	100% / 0% / 0%
13	X-Golden Retriever	12	female	spay	29.5	0% / 100% / 0%	1% / 99% / 0%
14	West Highland White Terrier	6	male	neutered	NA	0% / 100% / 0%	100% / 0% / 0%
15	mixed breed	9	male	neutered	16.3	0% / 100% / 0%	0% / 100% / 0%
16	Chihuahua	6	male	intact	3.5	0% / 100% / 0%	100% / 0% / 0%
17	X-Terrier	11	male	neutered	9.4	0% / 100% / 0%	80% / 20% / 0%
18	Schnauzer, Miniature	9	male	neutered	9.9	0% / 100% / 0%	100% / 0% / 0%
19	X-Welsh Corgi	11	male	neutered	14.8	100% / 0% / 0%	100% / 0% / 0%
20	Cairn Terrier	11	female	spay	8.0	90% / 10% / 0%	100% / 0% / 0%
21	Pug	8	male	neutered	10.8	0% / 100% / 0%	(hollow)
22	Havanese	9	male	neutered	5.4	100% / 0% / 0%	100% / 0% / 0%
23	Pomeranian	7	male	intact	4.2	100% / 0% / 0%	100% / 0% / 0%
24	X-Labrador Retriever	11	male	neutered	36.0	100% / 0% / 0%	100% / 0% / 0%
25	X-Schnauzer, Miniature	11	female	spay	7.8	100% / 0% / 0%	100% / 0% / 0%
26	Yorkshire Terrier	10	male	neutered	6.2	0% / 100% / 0%	100% / 0% / 0%
27	Siberian Husky	13	male	neutered	19.6	0% / 100% / 0%	99% / 1% / 0%
28	X-Pit Bull	3	male	neutered	NA	0% / 100% / 0%	100% / 0% / 0%
29	X-Chihuahua	4	male	neutered	8.2	0% / 100% / 0%	100% / 0% / 0%
30	Shih Tzu	7	female	spay	6.4	0% / 100% / 0%	99% / 1% / 0%
31	X-Border Terrier	6	female	spay	12.3	1% / 99% / 0%	100% / 0% / 0%
32	X-Shih Tzu	10	male	neutered	9.5	100% / 0% / 0%	100% / 0% / 0%
33	X-Pomeranian	7	male	neutered	7.7	0% / 100% / 0%	100% / 0% / 0%
34	Husky	10	male	neutered	28.0	5% / 95% / 0%	100% / 0% / 0%
35	Bull Terrier	8	male	neutered	31.4	0% / 100% / 0%	100% / 0% / 0%
36	Pit Bull	9	male	neutered	33.0	0% / 100% / 0%	100% / 0% / 0%
37	X-Bichon Frise	14	male	neutered	6.6	20% / 80% / 0%	100% / 0% / 0%
38	Pomeranian	NA	male	intact	5.6	100% / 0% / 0%	100% / 0% / 0%
39	Havanese	9	female	spay	7.3	100% / 0% / 0%	100% / 0% / 0%
40	Lhasa Apso	NA	male	neutered	11.1	89% / 10% / 1%	100% / 0% / 0%
41	X-Schipperke	11	male	neutered	5.7	0% / 100% / 0%	5% / 94% / 1%
42	X-Yorkshire Terrier	9	male	neutered	NA	10% / 90% / 0%	100% / 0% / 0%
43	X-Cocker Spaniel	12	male	neutered	9.8	20% / 80% / 0%	100% / 0% / 0%
44	mixed breed	12	male	neutered	7.3	0% / 100% / 0%	99% / 1% / 0%
45	Border Collie	8	male	neutered	29.0	100% / 0% / 0%	100% / 0% / 0%
46	X-Terrier	12	male	neutered	NA	30% / 70% / 0%	100% / 0% / 0%
47	French Bulldog	13	female	spay	8.4	0% / 100% / 0%	100% / 0% / 0%
48	Chihuahua	4	male	intact	2.0	1% / 99% / 0%	100% / 0% / 0%
49	X-Poodle	11	male	neutered	10.1	25% / 75% / 0%	100% / 0% / 0%
50	Chihuahua	5	male	neutered	7.2	100% / 0% / 0%	100% / 0% / 0%
51	Schnauzer, Miniature	11	male	neutered	8.8	50% / 50% / 0%	100% / 0% / 0%
52	Schnauzer, Miniature	8	male	neutered	8.1	1% / 99% / 0%	100% / 0% / 0%
53	X-Chihuahua	11	male	neutered	7.3	100% / 0% / 0%	100% / 0% / 0%

The description of the dogs and associated data are listed. For breed, a single breed name indicates a purebred canine as reported by the owner. An “X-”preceding breed name indicates a mixed breed, with the subsequent breed name indicating the most dominant breed features of the non-purebred as reported by the owner. A listing of “mixed breed” indicates that no dominant breed characteristic pattern was observable. Age at time of stone removal is listed for most dogs, except 2 listed as “*NA*” indicating no data was available. “Status” category indicates whether the reproductive capacity of the dog was intact or spay/neutered. “Weight” category indicates weight of dog in kg at time of urinary stone collection. Composition information for both the outer surface and inner core regions of the stones is reported, with percentage of COM (% COM), COD (% COD), and apatite (% APA).

The wide range of some elements within the canine stones raised the possibility of associations with other characteristics of the stones or whole animal (Table 2 **)**. There was no significant correlation between Ca levels in stones to age of dog at stone removal, sex, and reproductive status of animal, weight of animal, and hydration type of CaOx mineral (COM vs. COD). Furthermore, no significant correlations were found between consumption of dry compared to canned food and Ca levels in the canine stones (not shown). Qualitative evaluation of Ca levels in canine stones to specific breeds or specific diet manufacturers revealed no obvious association with the extreme Ca values (not shown). However, significant associations between P and reproductive status of male animals was noted, specifically that greater P was found in stones from intact compared to neutered male canines (Table 2). This relationship was not measurable in females because only 1 female dog was intact in this sample of stone-forming canines. Comparison of P levels in canine stones to all other factors revealed no significant correlations. Also, the levels of B, Ba, Cr, and Zn were found to be significantly more abundant in intact male canines compared to neutered male canines. There was an apparent trend for higher Cu and Mg levels in intact male canines compared to neutered male canines, but did not reach significance. Additionally, the levels of K, Mg, and S were found to be significantly more abundant in COM than in COD mineral types.

**Table 2:** Association analysis of stone composition with characteristics of canines and stones.

ELEMENT	AGE OF DOG (r2)	SEX (p)	MALE STATUS (p)	FOOD TYPE (p)	STONE SURFACE (p)
**Ca**	0.01	0.4777	0.5595	0.3587	0.9260
**P**	0.00	0.1868	0.0028	0.7573	0.1869
**Na**	-0.03	0.5301	0.1193	0.0927	0.2925
**Mg**	0.00	0.4175	0.0862	0.2278	0.0003
**K**	0.02	0.6759	0.2582	0.2483	0.0001
**S**	0.03	0.1868	0.6681	0.9434	0.0001
**Sr**	0.01	0.1356	0.8722	0.1888	0.2243
**Zn**	-0.04	0.5301	0.0001	0.6688	0.2925
**Fe**	-0.04	0.8967	0.4913	0.2699	0.1097
**Si**	0.11	0.8397	0.7126	0.1194	0.6693
**Cu**	0.09	0.1019	0.0576	0.3730	0.4102
**Al**	-0.01	0.1057	0.1792	0.9609	0.4740
**Ba**	0.00	0.8177	0.0099	0.2271	0.1342
**Mo**	-0.50	NA	NA	NA	NA
**Pb**	0.00	0.5238	0.2945	0.7967	0.8716
**B**	-0.04	0.2062	0.0019	0.7387	0.6338
**V**	0.00	NA	0.5230	0.5264	0.4107
**Cr**	0.00	0.7288	0.0344	0.7311	0.1288
**Mn**	0.02	0.3361	0.2208	0.5149	0.1522

The elemental content of the canine stones was compared to physical (age, sex, and reproductive status) or dietary characteristics of the dog. The elemental content of the canine stones was also compared to mineral type of each stone. The linear regression function between element content in canine stone and dog age at time of stone collection was evaluated using the coefficient of determination (r^2^). The remaining categories were tested by standard t-test between element content in stone and category value. “Sex” category indicates whether dog was male or female, regardless of reproductive capacity. “Male Status” category indicates whether male dog was intact or neutered; female dogs were not compared due to few stones from intact female dogs. “Food Type” category indicates whether dog was predominantly fed commercial dry pellets or canned food. “Stone Surface” category indicates whether the surface of the canine stone was determined to be predominantly (>99%) COM or COD; stones with mixed mineral types were not included in this analysis. The mineral type at the core of the canine stone was not compared because most (>90%) stones had cores containing COM, regardless of the surface mineral type. A value of “p<0.05” indicates that the specific element significantly correlated with a specific value of the category. “*NA*” indicates that too few (n<3) points were available for comparison for the specific element and category value.

The similarity in associations among several elements – particularly Mg, P, and Zn – prompted an inquiry as to whether specific elemental content would correlate with other elements within the canine CaOx stone. To determine if there were elements that associated with each other within the stones, the levels of each element levels was plotted against all other elements and then evaluated by a linear regression function; the results of this analysis are displayed in a correlation matrix (Table 3). Significance was determined by the r^2^ of the linear regression function, with values between +/-0.2 considered non-significant. The strongest positive correlations (r^2^ > 0.6) were found between the element pairs of Mg/K, Mg/Na, Mg/P, P/B, P/Ba, P/Cr, and P/Zn. The strongest negative correlations were found between Mo and the elements Ba, Pb, and Zn, though the strength of the association was significantly lower than the positive associations.

**Table 3:** Correlation matrix of element content within canine calcium oxalate-based urinary stones.

	Al	B	Ba	Ca	Cr	Cu	Fe	K	Mg	Mn	Mo	Na	P	Pb	S	Si	Sr	V	Zn
**Al**	1																		
**B**	0.02	1																	
**Ba**	0.16	0.35	1																
**Ca**	0.40	0.00	0.01	1															
**Cr**	0.15	0.45	0.54	0.40	1														
**Cu**	-0.01	-0.08	0.00	-0.01	-0.02	1													
**Fe**	-0.01	-0.18	0.00	0.01	0.00	0.00	1												
**K**	0.13	0.06	0.30	0.07	0.37	0.00	0.00	1											
**Mg**	0.05	0.18	0.41	0.00	0.38	0.00	0.00	0.69	1										
**Mn**	0.02	-0.02	0.10	0.49	0.18	-0.07	0.09	0.31	0.06	1									
**Mo**	-0.17	0.00	-0.25	0.02	NA	NA	-0.01	0.09	0.00	NA	1								
**Na**	0.00	0.18	0.19	0.00	0.24	0.00	0.00	0.42	0.68	0.01	0.04	1							
**P**	0.08	0.60	0.60	0.03	0.67	0.00	-0.02	0.35	0.61	0.03	-0.11	0.46	1						
**Pb**	0.00	0.02	0.08	0.01	0.45	-0.01	-0.06	0.01	0.07	-0.03	-0.23	0.03	0.11	1					
**S**	0.00	0.00	-0.01	0.04	0.06	0.00	0.14	0.34	0.13	0.22	0.53	0.16	0.01	-0.01	1				
**Si**	0.09	0.09	-0.04	0.01	-0.02	-0.11	0.01	0.08	0.01	0.02	NA	-0.05	-0.04	-0.02	0.00	1			
**Sr**	0.03	0.00	0.06	0.15	0.12	0.01	0.00	0.12	0.10	0.54	-0.06	0.13	0.04	0.01	0.01	0.06	1		
**V**	0.02	0.09	0.03	0.02	0.01	0.01	-0.01	0.01	0.02	-0.13	NA	0.01	0.13	0.04	-0.05	-0.05	-0.02	1	
**Zn**	0.40	0.51	0.40	0.04	0.48	0.00	0.00	0.11	0.26	-0.03	-0.21	0.12	0.64	0.08	0.00	-0.08	0.01	0.02	1

The matrix displays the coefficient of determination (r^2^) of the linear regression function between each pair of elements measured within the canine urinary stone samples. “*NA*” indicates that too few (n<6) points were available for high confidence comparison for the specific element pair. Values between +/- 0.2 were considered non-significant. Values greater than +0.6 or less than -0.6 were considered highly significant.

**Fig 2:**
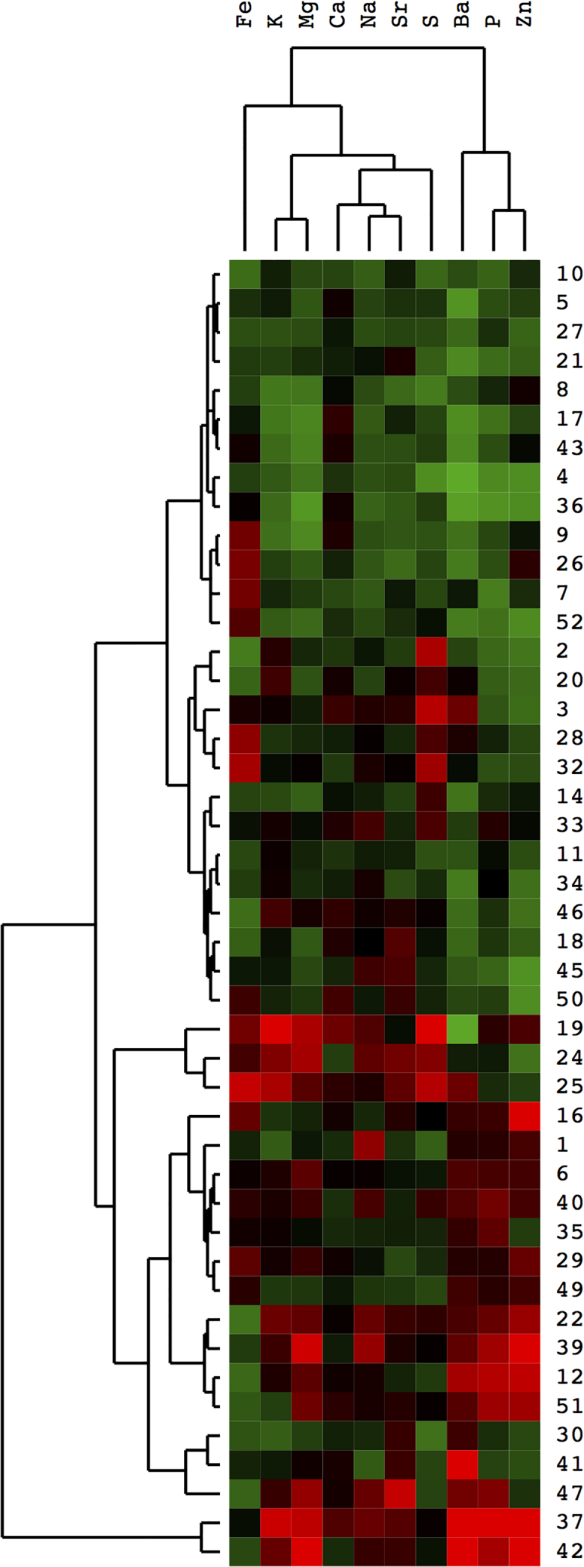
Cluster analysis of elemental content within canine calcium oxalate-based urinary stones. Weight-normalized values for each elemental group were log-transformed and normalized to the group mean before being entered into a hierarchical clustering model with correlation defined by a Euclidean distance function. Elemental groups with missing data were removed, leaving 10 elements for the analysis. The heatmap displays the relative divergence from the mean with increasing shades of red being higher and increasing shades of green being lower than mean value of the elemental group. The numbers on the side of the heatmap identify the specific canine stone sample, whereas the elemental category is described across the top of the heatmap. The dendrogram displays the different clades, with greater height of the branch points indicating greater differences between the leaves.

Cluster analysis was then utilized to find associated groups of elements within the canine CaOx stones. A subset of element values were log-transformed, normalized to the group mean, and entered into an unsupervised hierarchical clustering model with correlation defined by a Euclidean distance function. The results were then visualized using heatmap and dendrogram functions (Fig 2). Among the elemental clades, the grouping that defines the greatest differences within the canine stones was the combination of Ba, P, and Zn. This was an interesting finding, as the stone content of Ba, P, and Zn had demonstrated association with each other and with specific stone and whole animal characteristics in previous correlation analyses. Within the next set of elemental clades, the Fe content was the next most important defining variable, followed by the group of K and Mg. Ca was among the least important elements in defining new clades, despite the abundance of Ca levels in the stones. Additionally, the canine stones themselves were clustered based on their elemental profile. The most significant clade contained only two stones, which had high values for most elements; since this was a rare phenotype, so we did not focus on this result. The second-most significant clade was based predominantly on higher levels of the group Ba, P, and Zn, which accounted for about 40% of the samples; the other 60% mostly had lower-than-mean levels of these 3 elements. This result was interesting because it suggested a subgroup of canine CaOx stone types that had not previous been reported. The next most significant clade was again based on high Fe, K, and Mg values, reflecting what had been observed with the elemental grouping results.

## Discussion

Urinary stone formation often occurs spontaneously in the dog and human, but the underlying causes are not understood. CaOx has become the most common mineral detected in canine uroliths, as in humans [3,27]. Dogs also share many risk factors for stone disease with humans, such as being male and overweight [3,27–30]. These similarities suggest that the dog might serve as a model for urinary stone disease to help improve outcomes in both species. Comparison of the median and range of elemental content for the canine stones revealed a high degree of overlap with the median and range of most elements reported for human stones from all known reports published in the last 5 years. Overall, these findings support a conclusion that the canine CaOx stone is chemically similar to the analogous human stones at the inorganic level. Further work looking at organic and structural components would be helpful to confirm the similarities in the animal model.

In the canine stones, the most abundant metal was Ca at 215 ± 53 mg per gram stone, or over 20% by mass. The Ca content in pure COM is 27% and COD is 24%, but actual stones (canine and human) are known to also contain small amounts of protein, small molecules, and other metals [11–16], so it is expected that actual Ca levels in CaOx stones would be less than the theoretical estimates. Following Ca are the constitutive elements (<1000 μg/g stone) P, Na, Mg, K, and S, which are known to be present in biomineral formations. Phosphate minerals (e.g. brushite and apatite) are often found within Ca-based stones, so high P levels were expected in these stones [31]. The levels of Na, Mg, and K are high in the urine, so the presence of these metals in canine stones not surprising. S is expected as well, as sulfates and proteins are also known to be in urinary stones. The next set of elements detected in the canine stones was the trace elements (10–1000 μg/g stone), which include Sr, Zn, and Fe. Many of these metals are known to be present in urinary stones, especially Sr and Zn that have similar physical properties to Ca and have been shown to affect stone properties [23,32–35]. Finally, several ultratrace elements (<10 μg/g stone) were detected in the canine stones, such as Ba, B, and Mn. Some of the ultratrace elements have been measured in previous studies of stone composition [23], although all remain poorly characterized.

The elemental content in canine CaOx stones was compared with other characteristics of the stone or whole animal, in line with previous studies [27,36]. Ca levels did not demonstrate any significant relationship with characteristics of stone or animal. The standard deviation of Ca levels between canine stones was only approximately 25% of the group median, indicating a low amount of variance in Ca levels within the stones. Thus any difference in Ca between subgroups of CaOx stones would be small. Conversely, P had a wider standard deviation of 70% from the group median, indicating greater variance. When tested for associations with dog or stone characteristics, P content was significantly greater in stones from intact male canines compared to neutered male canines. This pattern was found to be the same for Zn content in the canine stones, and a trend with Mg content. It is unclear why canine stones from intact males would be greater in P and Zn. The intact males would likely have higher circulating androgen hormones, which can affect circulating and tissues levels of Zn, but there are no reports on androgen levels and stone Zn levels.

Another association was observed for the mineral type of the surface of the canine CaOx stones. These stones containing COM were found to have significantly elevated levels of Mg and S compared to stones mostly containing COD. The different CaOx types belong to distinct crystal systems (COM is monoclinic while COD is tetragonal), so the crystal type may affect the inclusion rates for other elements. Mg content has been measured in bone and urinary stones of mammals, and is known to be able to compete for binding to Ca binding sites, including oxalate [37]. Molecular dynamics studies showed that the presence of Mg alters the CaOx mineral structure by reducing the size of the crystal aggregates and destabilizing ionic pairing [38]. Moreover, it has been reported in human patients that lower levels of Mg in stones composed primarily of COM were associated with the stones being more resistant to SWL [19]. In this study, Mg levels in canine CaOx stones had a standard deviation of 70% from the group median. However, the effect of Mg levels on canine stone hardness is not known. S is also abundant in the canine CaOx stone, but less is known about the form and role of S compared to Mg. S, as sulfate, can compete for Ca binding, and has been proposed to affect stone formation rates in humans, but S could also be present as S-containing amino acids or small molecules like thioesters [39]. It is difficult to propose a role for S within the canine stones without knowing the major form(s).

Element-element relationships were also evaluated in the canine CaOx stones, as has been reported in previous human stone studies [34,40]. Ca failed to demonstrate significant correlations with the other elements. The strongest positive correlations of element pairs were found among the elements B, Ba, Cr, K, Mg, Na, P, and Zn (r^2^ >0.6). In this group of elements, Mg and P had the greatest number of associations, with participation in more than 3 significant correlation pairings. As described previously, Mg content in human stones has been proposed to affect urinary stone formation and hardness, but further study is needed in canine stones. P levels in stones is even less understood than Mg, but thought to be important as phosphate minerals may affect stone formation. The strongest negative correlations were found between Mo in a correlation pair with the elements Ba, Pb, or Zn, although the strength of the correlation was lower than with the positive correlations. There is little information as to the role of Mo within uroliths [16].

Since several of the same elements correlated with stone characteristics and/or each other, hierarchical cluster analysis was used to determine the strength of grouping effects with these elements in the canine CaOx stones. The results indicated that Ba, P, and Zn were a significant grouping set of elements that defined some of the most significant differences within the stones, leading to natural subgroups based solely on elemental content. This was an interesting finding, since Ba, P, and Zn had some of the stronger associations among the collective constituent elements. Secondary grouping within the primary element divisions was driven by the elements Fe, K, and Mg. The importance of these subgroups is not immediately clear, but these groupings may correlate with etiological or physical properties of the stone. For example, the pattern of high Ba, P, and Zn content may strongly predict a stone that is resistant to SWL, which may influence the best clinical treatment strategy.

Ultimately, what drives the elemental content within the canine urinary stones is not clear. Historically, patients were advised to avoid Ca-containing foods to reduce Ca-based stone formation. However, this approach has since been discredited, as epidemiological and a few prospective studies in humans failed to show a benefit of reduction of dietary Ca, unless the baseline Ca intake was very high [41–43]. We now understand that urinary Ca is highly regulated, and dietary changes are poorly effective in changing urinary Ca excretion in patients. In dogs, the effect of Ca intake on Ca-based stone risk has lead to mixed results in a few epidemiological and prospective studies [44–46]. The effects of trace metal intake are not understood for either canine or human CaOx stone risk. For Fe and Zn, the renal system is not the dominant route of excretion, so little is known about the renal handling of these metals. However, it is possible that dietary manipulation could have a supportive role in the prevention of stones if it were shown that any of the non-calcium metals have a role in urolithogensis. In dogs, specialty foods marketed for stone prevention are available to the consumer, but the Ca and other components are not well standardized. If specific minerals associate with relevant stone properties, changes in canine diet formulation may be warranted.

## Supporting Information

S1 Table Element wavelengths and limits used for elemental analysis. The listed elements at given wavelength (nm) were chosen for optimal analytic characteristics for analysis of CaOx stones. The minimum detection limit for each element is listed (ppm). The maximum detection limit is not applicable as all samples were diluted until the queried element was within detection range of the instrument calibration.
(DOCX)

S2 Table Weight-normalized elemental content in canine calcium oxalate-type urinary stones. The elemental content (μg element/g stone) content of 19 elements (n = 6–53) quantified from CaOx stones types from canine patients is listed and ordered based on abundance based on group median, as illustrated in **Fig 1**. Data includes the group median, the maximum and minimum values, and the interquartile range (25% and 75%). Additional data includes the “%CV,” the coefficient of variation (standard deviation/mean), and “kurtosis,” fourth moment of the population, as measures of frequency distribution of the elemental values.
(DOCX)

## References

[1] Ramello A, Vitale C, Marangella M. J Nephrol. 2000 Nov-Dec;13 Suppl 3:S45–50. 11132032

[2] Bagga HS, Chi T, Miller J, Stoller ML. New insights into the pathogenesis of renal calculi. Urol Clin North Am. 2013 2;40(1):1–12. 10.1016/j.ucl.2012.09.006 23177630 PMC4165395

[3] Low WW, Uhl JM, Kass PH, Ruby AL, Westropp JL. Evaluation of trends in urolith composition and characteristics of dogs with urolithiasis: 25,499 cases (1985–2006). J Am Vet Med Assoc. 2010 1 15;236(2):193–200. 10.2460/javma.236.2.193 20074011

[4] Lulich JP, Osborne CA, Lekcharoensuk C, Kirk CA, Allen TA. Effects of hydrochlorothiazide and diet in dogs with calcium oxalate urolithiasis. J Am Vet Med Assoc. 2001 5 15;218(10):1583–6. 11393368 10.2460/javma.2001.218.1583

[5] Stevenson AE, Blackburn JM, Markwell PJ, Robertson WG. Nutrient intake and urine composition in calcium oxalate stone-forming dogs: comparison with healthy dogs and impact of dietary modification. Vet Ther. 2004 Fall;5(3):218–31. 15578454

[6] Adams L.G., Berent A.C., Moore G.E. Bagley DH. Use of laser lithotripsy for fragmentation of uroliths in dogs: 73 cases (2005–2006). J Am Vet Med Assoc. 2008 232, 11 1680–1687. 10.2460/javma.232.11.1680 18518810

[7] Osborne CA, Lulich JP, Forrester D, Albasan H. Paradigm changes in the role of nutrition for the management of canine and feline urolithiasis. Vet Clin North Am Small Anim Pract. 2009 1;39(1):127–41. 10.1016/j.cvsm.2008.10.001 19038655

[8] Dijcker JC, Hagen-Plantinga EA, Everts H, Bosch G, Kema IP, Hendriks WH. Dietary and animal-related factors associated with the rate of urinary oxalate and calcium excretion in dogs and cats. Vet Rec. 2012 7 14;171(2):46 10.1136/vr.100293 22735988

[9] Arulpragasam SP, Case JB, Ellison GW. Evaluation of costs and time required for laparoscopic-assisted versus open cystotomy for urinary cystolith removal in dogs: 43 cases (2009–2012). J Am Vet Med Assoc. 2013 9 1;243(5):703–8 10.2460/javma.243.5.703 23971851

[10] Furrow E, Patterson EE, Armstrong PJ, Osborne CA, Lulich JP. Fasting Urinary Calcium-to-Creatinine and Oxalate-to-Creatinine Ratios in Dogs with Calcium Oxalate Urolithiasis and Breed-Matched Controls. J Vet Intern Med. 2015 1;29(1):113–9. 10.1111/jvim.12527 25581880 PMC4311896

[11] Nagy Z, Szabo E, Kelenhei M. Spectrum analysis of kidney calculi for metal trace elements. Zeitschrift fur Urologie. 1963;56:186–90. 13937198

[12] Fleisch H. Inhibitors and promoters of stone formation. Kidney Int. 1978 5;13(5):361–71 351264 10.1038/ki.1978.54

[13] Durak I, Yasar A, Yurtarslani Z, Akpoyraz M, Tasman S. Analysis of magnesium and trace elements in urinary calculi by atomic absorption spectrophotometry. Br J Urol. 1988 9;62(3):203–5. 3191333 10.1111/j.1464-410x.1988.tb04318.x

[14] Atmani F, Glenton PA, Khan SR. Identification of proteins extracted from calcium oxalate and calcium phosphate crystals induced in the urine of healthy and stone forming subjects. Urol Res. 1998;26(3):201–7. 9694603 10.1007/s002400050047

[15] Canales BK, Anderson L, Higgins L, Ensrud-Bowlin K, Roberts KP, Wu B, et al Proteome of human calcium kidney stones. Urology. 2010 10;76(4):1017 10.1016/j.urology.2010.05.012 20709378

[16] Słojewski M. Major and trace elements in lithogenesis. Cent European J Urol. 2011;64(2):58–61. 10.5173/ceju.2011.02.art1 24578864 PMC3921713

[17] Dretler SP. Stone fragility—a new therapeutic distinction. J Urol. 1988 5;139(5):1124–7. 3361657 10.1016/s0022-5347(17)42801-1

[18] Madaan S, Joyce AD. Limitations of extracorporeal shock wave lithotripsy. Curr Opin Urol. 2007 3;17(2):109–13. 17285020 10.1097/MOU.0b013e32802b70bc

[19] Turgut M, Unal I, Berber A, Demir TA, Mutlu F, Aydar Y. The concentration of Zn, Mg and Mn in calcium oxalate monohydrate stones appears to interfere with their fragility in ESWL therapy. Urological Research. 2008;36(1):31–8. 10.1007/s00240-007-0133-1 18176803

[20] El Prien, Frondel C. Studies in urolithiasis; the composition of urinary calculi. J Urol. 1947 6;57(6):949–94. 20243909 10.1016/S0022-5347(17)69732-5

[21] Motulsky HM, Brown RE. Detecting outliers when fitting data with nonlinear regression – a new method based on robust nonlinear regression and the false discovery rate. BMC Bioinformatics 2006, 7:123 16526949 10.1186/1471-2105-7-123 PMC1472692

[22] Eisen MB, Spellman PT, Brown PO, Botstein D. Cluster analysis and display of genome-wide expression patterns. Proc Natl Acad Sci U S A. 1998 12 8;95(25):14863–8. 9843981 10.1073/pnas.95.25.14863 PMC24541

[23] Słojewski M, Czerny B, Safranow K, Jakubowska K, Olszewska M, Pawlik A, et al Microelements in stones, urine, and hair of stone formers: a new key to the puzzle of lithogenesis? Biol Trace Elem Res. 2010 12;137(3):301–16. 10.1007/s12011-009-8584-6 20024629

[24] Giannossi ML, Summa V, Mongelli G. Trace element investigations in urinary stones: a preliminary pilot case in Basilicata (Southern Italy). J Trace Elem Med Biol. 2013 4;27(2):91–7. 10.1016/j.jtemb.2012.09.004 23141501

[25] Abdel-Gawad M, Ali-El-Dein B, Mehta S, Al-Kohlany KM, Elsobky E. A correlation study between macro- and micro-analysis of pediatric urinary calculi. J Pediatr Urol. 2014 12;10(6):1267–72. 10.1016/j.jpurol.2014.06.022 25155408

[26] Keshavarzi B, Yavarashayeri N, Irani D, Moore F, Zarasvandi A, Salari M. Trace elements in urinary stones: a preliminary investigation in Fars province, Iran. *Environ Geochem Health*. 2014 Nov 30. 10.1007/s10653-014-9654-z 25433503

[27] Ling GV, Thurmond MC, Choi YK, Franti CE, Ruby AL, Johnson DL. Changes in proportion of canine urinary calculi composed of calcium oxalate or struvite in specimens analyzed from 1981 through 2001. J Vet Intern Med. 2003 Nov-Dec;17(6):817–23. 14658718 10.1111/j.1939-1676.2003.tb02520.x

[28] Lekcharoensuk C, Lulich JP, Osborne CA, Pusoonthornthum R, Allen TA, Koehler LA, et al Patient and environmental factors associated with calcium oxalate urolithiasis in dogs. J Am Vet Med Assoc. 2000 8 15;217(4):515–9. 10953715 10.2460/javma.2000.217.515

[29] Houston DM, Moore AE, Favrin MG, Hoff B. Canine urolithiasis: a look at over 16 000 urolith submissions to the Canadian Veterinary Urolith Centre from February 1998 to April 2003. Can Vet J. 2004 Mar;45(3):225–30. 15072194 PMC548608

[30] Wisener LV, Pearl DL, Houston DM, Reid-Smith RJ, Moore AE. Risk factors for the incidence of calcium oxalate uroliths or magnesium ammonium phosphate uroliths for dogs in Ontario, Canada, from 1998 to 2006. Am J Vet Res. 2010 9;71(9):1045–54. 10.2460/ajvr.71.9.1045 20807144

[31] Tiselius HG, Larsson L. Calcium phosphate: an important crystal phase in patients with recurrent calcium stone formation? Urol Res. 1993 5;21(3):175–80. 8342251 10.1007/BF00590033

[32] Tang Y, Chappell HF, Dove MT, Reeder RJ, Lee YJ. Zinc incorporation into hydroxylapatite. Biomaterials. 2009 5;30(15):2864–72. 10.1016/j.biomaterials.2009.01.043 19217156

[33] Blaschko SD, Chi T, Miller J, Flechner L, Fakra S, Kapahi P, et al Strontium substitution for calcium in lithogenesis. J Urol. 2013;189(2):735–9. 10.1016/j.juro.2012.08.199 23260568 PMC4124908

[34] Durak I, Kilic Z, Sahin A, Akpoyraz M. Analysis of calcium, iron, copper and zinc contents of nucleus and crust parts of urinary calculi. Urol Res. 1992;20(1):23–6. 1736483 10.1007/BF00294330

[35] Atakan IH, Kaplan M, Seren G, Aktoz T, Gül H, Inci O. Serum, urinary and stone zinc, iron, magnesium and copper levels in idiopathic calcium oxalate stone patients. Int Urol Nephrol. 2007;39(2):351–6. 17203355 10.1007/s11255-006-9050-4

[36] Osborne CA, Lulich JP, Polzin DJ, Sanderson SL, Koehler LA, Ulrich LK, et al Analysis of 77,000 canine uroliths. Perspectives from the Minnesota Urolith Center. Vet Clin North Am Small Anim Pract. 1999;29(1):17–38. 10028149 10.1016/s0195-5616(99)50002-8

[37] Romani AM. Cellular magnesium homeostasis. Arch Biochem Biophys. 2011 8 1;512(1):1–23. 10.1016/j.abb.2011.05.010 21640700 PMC3133480

[38] Riley JM, Kim H, Averch TD, Kim HJ. Effect of Magnesium on Calcium and Oxalate Ion Binding. J Endourol. 2013;27(12):1487–92. 10.1089/end.2013.0173 24127630 PMC3883082

[39] Rodgers A, Gauvin D, Edeh S, Allie-Hamdulay S, Jackson G, Lieske JC. Sulfate but not thiosulfate reduces calculated and measured urinary ionized calcium and supersaturation: implications for the treatment of calcium renal stones. PloS One. 2014;9(7):e103602 10.1371/journal.pone.0103602 25061988 PMC4111609

[40] Fang X, Ahmad SR, Mayo M, Iqbal S. Elemental analysis of urinary calculi by laser induced plasma spectroscopy. Lasers Med Sci. 2005;20(3–4):132–7. 16193228 10.1007/s10103-005-0356-8

[41] Curhan GC, Willett WC, Rimm EB, Stampfer MJ. A prospective study of dietary calcium and other nutrients and the risk of symptomatic kidney stones. N Engl J Med. 1993;328(12):833–8. 8441427 10.1056/NEJM199303253281203

[42] Lewandowski S, Rodgers AL. Idiopathic calcium oxalate urolithiasis: risk factors and conservative treatment. Clin Chim Acta. 2004 7;345(1–2):17–34. 15193974 10.1016/j.cccn.2004.03.009

[43] Heilberg IP, Goldfarb DS. Optimum nutrition for kidney stone disease. Adv Chronic Kidney Dis. 2013;20(2):165–74. 10.1053/j.ackd.2012.12.001 23439376

[44] Lekcharoensuk C, Osborne CA, Lulich JP, Pusoonthornthum R, Kirk CA, Ulrich LK, Koehler LA, Carpenter KA, Swanson LL. Associations between dry dietary factors and canine calcium oxalate uroliths. Am J Vet Res. 2002;63(3):330–7. 11911566 10.2460/ajvr.2002.63.330

[45] Lekcharoensuk C, Osborne CA, Lulich JP, Pusoonthornthum R, Kirk CA, Ulrich LK, Koehler LA, Carpenter KA, Swanson LL. Associations between dietary factors in canned food and formation of calcium oxalate uroliths in dogs. Am J Vet Res. 2002 2;63(2):163–9. 11843112 10.2460/ajvr.2002.63.163

[46] Stevenson AE, Hynds WK, Markwell PJ. The relative effects of supplemental dietary calcium and oxalate on urine composition and calcium oxalate relative supersaturation in healthy adult dogs. Res Vet Sci. 2003;75(1):33–41. 12801461 10.1016/s0034-5288(03)00042-0

