# Supplementary material for: Elemental Content of Calcium Oxalate Stones from a Canine Model of Urinary Stone Disease
Source: PLoS One. 2015 Jun 11;10(6):e0128374. doi: 10.1371/journal.pone.0128374 (PMC4466234; doi:10.1371/journal.pone.0128374)
Supplement: S1 Table — The listed elements at given wavelength (nm) were chosen for optimal analytic characteristics for analysis of CaOx stones. The minimum detection limit for each element is listed (ppm). The maximum detection limit is not applicable as all samples were diluted until the queried element was within detection range of the instrument calibration. (DOCX) [file pone.0128374.s001.docx]

**Supplemental Table 1.** **Element wavelengths and limits used for elemental analysis**. The listed elements at given wavelength (nm) were chosen for optimal analytic characteristics for analysis of CaOx stones. The minimum detection limit for each element is listed (ppm). The maximum detection limit is not applicable as all samples were diluted until the queried element was within detection range of the instrument calibration.

| **ELEMENT** | **wavelength** | **minimum DL** |
| --- | --- | --- |
| Al | 394.401 | 0.313 |
| As | 188.980 | 0.313 |
| Au | 267.594 | 0.313 |
| B | 249.678 | 0.031 |
| Ba | 233.527 | 0.031 |
| Be | 234.861 | 0.031 |
| Ca | 373.690 | 0.313 |
| Cd | 214.439 | 0.031 |
| Co | 230.786 | 0.313 |
| Cr | 267.716 | 0.031 |
| Cu | 327.395 | 0.031 |
| Fe | 238.204 | 0.031 |
| In | 451.131 | 0.313 |
| K | 766.491 | 0.313 |
| Li | 610.365 | 0.313 |
| Mg | 280.270 | 0.313 |
| Mn | 259.372 | 0.031 |
| Mo | 202.032 | 0.313 |
| Na | 568.821 | 3.125 |
| Ni | 230.299 | 0.313 |
| P | 213.618 | 3.125 |
| Pb | 220.353 | 0.063 |
| Rb | 780.026 | 0.313 |
| S | 181.972 | 3.125 |
| Se | 196.026 | 0.313 |
| Si | 288.158 | 0.031 |
| Sn | 189.927 | 0.063 |
| Sr | 407.771 | 0.031 |
| Ti | 337.280 | 0.031 |
| Tl | 351.923 | 0.313 |
| V | 292.401 | 0.031 |
| Zn | 213.857 | 0.031 |
| Zr | 327.307 | 0.313 |
