# Supplementary material for: Elemental Content of Calcium Oxalate Stones from a Canine Model of Urinary Stone Disease
Source: PLoS One. 2015 Jun 11;10(6):e0128374. doi: 10.1371/journal.pone.0128374 (PMC4466234; doi:10.1371/journal.pone.0128374)
Supplement: S2 Table — The elemental content (μg element/g stone) content of 19 elements (n = 6–53) quantified from CaOx stones types from canine patients is listed and ordered based on abundance based on group median, as illustrated in Fig 1. Data includes the group median, the maximum and minimum values, and the interquartile range (25% and 75%). Additional data includes the “%CV,” the coefficient of variation (standard deviation/mean), and “kurtosis,” fourth moment of the population, as measures of frequency distribution of the elemental values. (DOCX) [file pone.0128374.s002.docx]

**Supplemental Table 2.** **Weight-normalized elemental content in canine calcium oxalate-type urinary stones**. The elemental content (µg element/g stone) content of 19 elements (n=6-53) quantified from CaOx stones types from canine patients is listed and ordered based on abundance based on group median, as illustrated in **Figure 1**. Data includes the group median, the maximum and minimum values, and the interquartile range (25% and 75%). Additional data includes the “%CV,” the coefficient of variation (standard deviation/mean), and “kurtosis,” fourth moment of the population, as measures of frequency distribution of the elemental values.

| **ELEMENT** | **minimum** | **25 Percentile** | **median** | **75 Percentile** | **maximum** | **%CV** | **kurtosis** |
| --- | --- | --- | --- | --- | --- | --- | --- |
| **Ca** | 127403.000 | 169984.000 | 210960.000 | 252933.000 | 355229.000 | 24.8 | -0.42 |
| **P** | 5792.000 | 15939.000 | 25225.000 | 46437.000 | 105267.000 | 69.5 | 1.37 |
| **Na** | 1925.000 | 2601.000 | 4305.000 | 6052.000 | 10443.000 | 45.1 | 0.22 |
| **Mg** | 574.400 | 1938.000 | 2990.000 | 5441.000 | 11443.000 | 69.6 | 0.69 |
| **K** | 626.800 | 1189.000 | 1716.000 | 2248.000 | 5669.000 | 56.6 | 2.09 |
| **S** | 153.600 | 465.400 | 608.000 | 971.500 | 2027.000 | 55.5 | 0.99 |
| **Sr** | 148.700 | 264.400 | 349.200 | 491.300 | 898.400 | 44.3 | 1.10 |
| **Zn** | 40.040 | 93.970 | 154.100 | 296.100 | 993.400 | 92.2 | 3.03 |
| **Fe** | 47.250 | 90.620 | 136.800 | 200.600 | 337.800 | 50.3 | -0.39 |
| **Si** | 0.867 | 2.714 | 12.960 | 72.460 | 156.000 | 122.1 | 0.27 |
| **Cu** | 1.443 | 4.526 | 8.288 | 16.990 | 84.270 | 113.1 | 5.81 |
| **Al** | 2.723 | 3.839 | 6.695 | 20.140 | 60.880 | 107.7 | 3.15 |
| **Ba** | 0.529 | 3.078 | 6.223 | 13.670 | 32.180 | 83.5 | 1.55 |
| **Mo** | 1.825 | 2.148 | 3.043 | 5.732 | 8.038 | 60.1 | 1.75 |
| **Pb** | 0.700 | 1.518 | 2.879 | 5.373 | 36.470 | 132.1 | 9.74 |
| **B** | 0.299 | 1.153 | 1.992 | 3.218 | 10.100 | 82.4 | 4.07 |
| **V** | 0.314 | 0.624 | 1.162 | 1.562 | 3.253 | 59.7 | 1.25 |
| **Cr** | 0.232 | 0.527 | 0.909 | 1.393 | 2.292 | 53.4 | 0.29 |
| **Mn** | 0.239 | 0.336 | 0.412 | 0.600 | 0.780 | 36.5 | -0.60 |
